# Differential influences of social support on app use for diabetes self-management – a mixed methods approach

**DOI:** 10.1186/s12911-020-01173-3

**Published:** 2020-07-07

**Authors:** Nicola Brew-Sam, Arul Chib, Constanze Rossmann

**Affiliations:** 1 Department of Health Services Research and Policy, Research School of Population Health, College of Health and Medicine, Australian National University, Mills Rd, Acton ACT 2601, Canberra, Australia; 2 Wee Kim Wee School of Communication and Information, Nanyang Technological University, 31 Nanyang Link, Singapore, 637718 Singapore; 3 Department of Media and Communication Studies, University of Erfurt, Nordhaeuser Str. 63, 99089 Erfurt, Germany

**Keywords:** mHealth, Apps, Technology, Diabetes, Self-management, Social support, Shared decision-making

## Abstract

**Background:**

Recent studies increasingly examine social support for diabetes self-management delivered via mHealth. In contrast to previous studies examining social support as an outcome of technology use, or technology as a means for delivering social support, this paper argues that social support has an impact on the use of diabetes mHealth apps. Specifically, we postulate differences between the impact of healthcare professional versus non-professional (family/friends) support on mobile app use for diabetes self-management.

**Methods:**

This research employed a triangulation of methods including exploratory semi-structured face-to-face interviews (*N* = 21, Study 1) and an online survey (*N* = 65, Study 2) with adult type 1 and type 2 diabetes patients. Thematic analysis (Study 1) was used to explore the relevance of social support (by professionals versus non-professionals) for diabetes app use. Binary logistic regression (Study 2) was applied to compare healthcare decision-making, healthcare-patient communication, and the support by the personal patient network as predictors of diabetes app use, complemented by other predictors from self-management and technology adoption theory.

**Results:**

The interviews (Study 1) demonstrated that (technology-supported) shared decision-making and supportive communication by healthcare professionals depended on their medical specialty. The personal patient network was perceived as either facilitating or hindering the use of mHealth for self-management. Binary logistic regression (Study 2) showed that the physician specialty significantly predicted the use of diabetes apps, with supervision by diabetes specialists increasing the likelihood of app use (as opposed to general practitioners). Additionally, specialist care positively related to a higher chance of shared decision-making and better physician-patient communication. The support by the personal patient network predicted diabetes app use in the opposite direction, with less family/friend support increasing the likelihood of app use.

**Conclusion:**

The results emphasize the relevance of support by healthcare professionals and by the patient network for diabetes app use and disclose differences from the existing literature. In particular, the use of diabetes apps may increase in the absence of social support by family or friends (e.g., compensation for lack of support), and may decrease when such support is high (e.g., no perceived need to use technology).

## Background

Prior research has suggested the impact of social support on diabetes management and health outcomes [1, 2]. With technological advances in healthcare, recent studies increasingly examine social support for diabetes self-management delivered through mobile online technologies (mHealth) (e.g. [3]). In this context, most studies considered social support as an outcome of mHealth use, or mHealth as a means for delivering social support. Applying a novel perspective to mHealth research, our paper investigates the influence of social support on the use of mHealth (apps), as implicated by technology adoption models.

In this paper, we distinguish between the impact of varied types of social support on diabetes self-management and outcomes in previous studies, further arguing that these sources of social support differentially influence technology-supported diabetes self-management. In particular, we identify and examine the varied influence of support by healthcare professionals and by the personal social patient network on the use of diabetes self-management apps. A separate study conducted on diabetes app quality and app features, as factors of interest, has been reported elsewhere [4, 5].

### The relevance of social support for diabetes self-management

Patient self-management, in which patients are decision-makers in control of their daily diabetes management [6], moved to the core of diabetes care when research revealed that patient non-compliance rates were high in care approaches based solely on healthcare professional responsibility (“doctor knows best principle”) [6]. Wilkinson, Whitehead, and Ritchie [7] reported that the ability to self-manage a diabetic condition (e.g., diet adherence, physical activity, medication intake, managing psychological aspects [8, 9]) is influenced by a whole series of factors (e.g., in [10, 11]). Prominently, social support has been reported to influence self-management behaviors and outcomes, associated with improved patient lifestyle and clinical outcomes, and reduced psychosocial disease symptoms [2]. The impact of support has been distinguished between that provided by healthcare professionals [12–14] and by the personal social patient network like relatives or peers [15–17]. This impact on diabetes management and health outcomes has been examined extensively, and been compared to one another ( [1, 18]; also see [4]).

First, studies on professional support show the beneficial effects of professional support on diabetes outcomes [19], especially when health professionals empower patients by using shared decision-making and supportive communication. A collaborative process of joint decision-making with an active contribution from both parties and the involvement of patients in treatment decisions (providing choices, considering patient preferences, and encouraging them to ask questions [13, 19, 20]) have been shown to be most supportive in disease care [21].

Second, in terms of non-professional support, family and friends can deliver emotional and practical support daily in diabetes care [22–25]. However, the personal social patient network has been reported to have a negative impact on diabetes self-management and health outcomes, when interactions like nagging behaviors, a lack in understanding, too much sympathy or pity expressed, or other negative behaviors occur [16, 26–29]. The concept of “miscarried helping” represents some of these problems [30].

### Social support and technology-supported diabetes self-management

Research has shown that interventions and care approaches need to differentially address support by the professional system and by the patient’s personal network for achieving improved outcomes [1]. This distinction applies similarly to research related to technology-supported diabetes care [31], which includes the use of diabetes apps that are designed to help patients improve their self-management (e.g., logbook apps).

In previous research, the use of (e.g., cloud-based) technology for support by healthcare professionals was reported to mostly result in positive outcomes for diabetes self-management [32–34]. Wehbe, Curcio, Gajjar, and Yadlapati [35] stated that the integration of technology into diabetes care affects physician-patient relationships positively when discussions are facilitated and when shared decision-making between physicians and patients is improved. However, according to these authors, the higher workload for physicians can also affect the relationship negatively. Abbasgholizadeh, Menear, Robitaille, and Legare [36] reported that health apps have potential for improved patient participation in shared health decision-making, but bear risks like security concerns or increased patient anxiety.

Beneficial effects of technology use on self-management and health outcomes were typically reported in studies that addressed non-professional social support by the patient’s personal networks [37, 38]. The use of social media, online communities, or mobile health (mHealth) has enhanced or improved psychological empowerment [39, 40], self-care information and knowledge [38, 41], blood glucose levels (HbA1c), as well as glucose self-monitoring and physical activity [3, 42], and other health aspects [43–45]. The inclusion of family members or spouses in technological diabetes management systems was found to improve their understanding of self-management requirements and affected the communication about diabetes and the support of the patient positively [46]. Notably, different studies reported that online social support by non-professionals might not always result in beneficial self-management and health outcomes. For example, this occurs when participation in online communities leads to mismatches between needed and received support, or when support hinders health improvements for patients with high self-efficacy [47].

### Research gap: the influence of (non-) professional social support on technology use

Existing studies almost exclusively focused on social support as an outcome of technology use, or on technology as a means for delivering social support. However, in order to increase an understanding of factors that influence technology use for self-care, research is still required that examines how professional and non-professional social support affect (mobile) diabetes technology use. It can be assumed that social support influences the use of mobile diabetes technology (e.g., apps) just as much as it influences traditional diabetes self-management behaviors (blood glucose testing, healthy nutrition, exercise, etc.) [4].

The number of studies examining factors influencing mHealth use is steadily rising [48], particularly in the context of disease self-management [49, 50]. While theoretical models of technology adoption [51] are one step towards understanding factors that influence (mobile) technology adoption and use, there is a gap in proving both significant and substantive usage effects for mHealth [52, 53]. Previous studies investigated factors influencing mHealth use in specific target groups (e.g. [54, 55]), for specific diseases or health topics [56–60], and for sustained mHealth engagement [61]. This study aims to explicate the effect of social support.

Social support has been shown to play a role as an influencing factor on aspects of technology use in mHealth studies. Zhang et al. [59], for instance, found that the theoretical factors of social influence and performance expectancy had the strongest direct influence on behavioral intention to use diabetes apps for disease management. Likewise, Quaosar et al. [55] found that social influence had a significant impact on intention to use mHealth, specifically in populations of higher age. We aim to address the research gap in these studies by comparing differential influences of healthcare professionals and personal patient networks on technology for self-care.

We hypothesize that both professional (e.g., physician) and non-professional support (e.g., family and friends) differentially influence diabetes app use (as a technology-supported self-management behavior). Following previous diabetes research it can be assumed that: *(H1) shared decision-making styles and supportive communication of healthcare professionals are promoting technology-supported self-management (app use), while (H2) the impact of support by the patient’s personal social network on technology (app) use for self-management depends on the type of perceived support (positive or misguided).*

## Methods

We used a mixed methods approach that included qualitative and quantitative data collection and analysis to permit “a more complete and synergistic utilization of data” ([62] , p.1). A detailed description of the methodology can be found in Brew-Sam [4, 63].

### Study 1: semi-structured face-to-face interviews

In the first study we conducted 21 semi-structured face-to-face interviews with type 1 (T1DM) and type 2 (T2DM) adult diabetes patients in Singapore. The interview guide [63] included questions on patients’ previous diabetes app (non-) use, their diabetes self-management, their attitudes, the support received from their medical team, relatives, and from others, as well as other relevant factors from self-management [7] and technology adoption theories, such as Unified Theory of Acceptance and Use of Technology (UTAUT) [51].

In addition to an interview guide developed based on these theories, we used a standardized background information questionnaire to collect relevant information about the patient background (e.g., demographic data, disease information) [63]. Open questions collected information about app and technology (non-) use as part of the diabetes self-management, as well as professional and non-professional social support, including shared decision-making in the patients’ treatment, their communication with healthcare professionals, and the support they received from their personal social network. Regarding the latter, we focused on family and friends’ support, being their strong-tie partners [64].

Ethics approval was sought and approved by the *Nanyang Technological University* Review Board (IRB-2016-01-012). Purposive sampling included patients with a variety of demographic and disease characteristics. Participants were informed about the study purpose and signed informed consent forms. All interviews were conducted by one researcher in English. The interviews were conducted and transcribed in 2015/2016 using the audio recording software “Audacity”. The audio files were completely and manually transcribed by three research assistants (explicit verbal content only); the cadence and cultural speech patterns have been retained for accuracy. The accuracy of transcription was checked, and names of interview participants were anonymized (interview participant/IP numbers).

Thematic analysis, as described by Braun and Clarke [65], was used for data analysis, following established standards for qualitative research [66]. A theme was defined as a topic resulting from different interview statements with similar content. First, preliminary categories were developed from the interviews regarding technology use, self-management, and social support using a matrix for data extracts. Broad themes were developed from the categories, which were reviewed against data extracts and the entire data set, before results were analyzed and interpreted (discussion among multiple researchers where results were ambiguous). A diabetes app (non-) user typology summarized the results on emerging themes and to map out differences in social support for different groups.

### Study 2: standardized online survey

In the second study we conducted a standardized online survey with 65 type 1 and type 2 diabetes patients. The questionnaire included questions on their diabetes background, self-management behaviors, mHealth use, social support, general attitudes and feelings, psychological empowerment, and demography [63].

Previous diabetes app use or non-use, as well as the length and frequency of previous app use were measured based on the National Survey on Health App Use [67]. Decision-making styles by health professionals and communication with the patient were measured using the Provider Participatory Decision-making Style Scale [13] (PDMstyle, *M* = 3.25, *SD* = .08, *α* = .943) and the Provider Communication Scale [13] (PCOM, *M* = 3.50, *SD* = .37, *α* = .919). Items from these scales, for example, asked to “specify how often the doctors or health care professionals who take care of your diabetes offered you choices in your medical care” (PDMstyle, “none of the time” to “all of the time”) and “tell us how the doctors or health care professionals who take care of your diabetes are at explaining treatment alternatives” (PCOM, “poor” to “excellent”). 5-point Likert scales were used throughout the survey. Social support by the personal social patient network was measured with a scale on support by family and friends from the Diabetes Care Profile (DCP, section V, *M* = 3.29, *SD* = .19, *α* = .931) [68, 69]. Scale items included questions on support delivered by family and friends, for example, “my family or friends help and support me a lot to take my medicine” (DCP, “strongly agree” to “strongly disagree”). Diabetes background and self-management data were adapted from the Diabetes Care Profile [69], two psychological empowerment scales [70, 71], the National Survey on Health App Use [67], and the Summary of Diabetes Self-Care Activities [72].

The survey was pre-tested with sixteen participants with and without diabetes. The online survey was disseminated (snowball method) to former face-to-face interview participants, as well as through social media and diabetes support groups. A lottery participation (monetary incentives: three coupons of 100 SGD each) was offered at the end of the survey.

Binary logistic regression (enter, blockwise) was used to test *H1* and *H2* on social support by health professionals versus non-professionals (family and friends) influencing diabetes app use, and to compare the strength of both (and other relevant included self-management and diabetes background factors from self-management and technology adoption theory) on diabetes app use. Independent variables were metric or coded as dummy variables, while diabetes app use was used as a binary variable (previous diabetes app use or non-use). Independent factors that included data from both app users and non-users were used in binary logistic regression. Regression models were compared for model fit, prediction success, and the inclusion of significant predictors by starting with all available factors, and then removing factors step by step.

### Study samples

Table 1 summarizes demographic and disease characteristics of the interview (Study 1, *N* = 21) and the online survey samples (Study 2, *N* = 65). Overall, the samples were diverse, including diabetes patients from various demographic and disease-related subgroups. The interview sample mostly included active patients managing their condition, with five respondents at high risk for health complications due to lacking diabetes knowledge, lacking motivation and insufficient self-management, unfavorable self-management attitudes, and/or critical health conditions (e.g., high blood glucose values). In both samples there were more type 2 diabetes patients than type 1 diabetes patients, following the global prevalence (~ 90%) of type 2 diabetes [73].

**Table 1:** Description of Study 1 and Study 2 Samples

	Study 1 – Interviews						Study 2 – Survey					
Variable	*n*	% of *N*	*M*	*SD*	*Min*	*Max*	*n*	% of *N*	*M*	*SD*	*Min*	*Max*
Age (in years)	–	–	48.35	17.46	19.00	68.00	–	–	49.74	14.67	20.00	70.00
Education												
MA graduate or higher	–	–					15	23.1				
BA graduate/college graduate	6	28.6					22	33.8				
Some college	7	33.3					12	18.5				
High school graduate	1	4.8					12	18.5				
Some high school	4	19.0					3	4.6				
Other education level	1	4.8					1	1.5				
Employment												
Full- time working	8	38.1					34	52.3				
Part-time working	3	14.3					12	18.5				
Homemaker	3	14.3					1	1.5				
Retired	3	14.3					7	10.8				
Student	3	14.3					5	7.7				
Unemployed	–	–					6	9.3				
Family status												
Married	9	42.9					32	49.2				
Never married	8	38.1					18	27.7				
Never married but relationship	–	–					4	6.2				
Separated/divorced	1	4.8					7	10.8				
Widowed	1	4.8					4	6.2				
Gender												
Men	11	52.4					32	49.2				
Women	10	47.6					33	50.8				
Nationality												
Singaporean	19	90.5					57	87.7				
Malaysian	1	4.8					4	6.2				
Other	1	4.8					4	6.2				
Diabetes Background												
Diabetes family history	12	57.1					50	76.9				
Diabetes type												
T2DM (incl. gestational)	11	52.4					50	76.9				
T1DM	9	42.9					13	20.0				
Pre-diabetes	1	4.8					2	3.1				
Diseases (other)	8	38.1					–	–				
Education on diabetes (received)	17	81.0					50	76.9				
Length of diabetes (in years)	–	–	19.89	12.07	4.00	38.00	–	–	13.73	9.81	.00	36.00
Medication												
Insulin injection (syringe or pump)	14	66.7					25	38.5				
Oral diabetes medication	10	47.6					51	78.5				
(Self-) Management												
Check-up frequency (in months)	–	–	3.83	1.36	2.00	6.00	–	–	–	–	–	–
Diabetes app use	11	52.4					–	–				
Never used	–	–					34	52.3				
Previous use	–	–					17	26.2				
Current use	–	–					14	21.5				
Online health information seeking (Study 2: days per week)	19	90.5	–	–	–	–	–	–	2.70	2.27	.00	7.00
Part of support group	15	71.4					–	–				
Offline	–	–					42	64.6				
Online	–	–					19	29.2				
Part of diabetes program	2	9.5					7	10.8				

*Note*. Table based on Brew-Sam [4]; Study 1: *N* = 21, Study 2: *N* = 65

## Results

### Interview results on social support and diabetes app use (Study 1)

As a foundation for comprehensively examining the influence of (*H1*) professional and (*H2*) non-professional support on diabetes app use, and to specify the hypotheses, themes were extracted from the interview data, relating to the aspects “previous app use for diabetes management”, “professional support”, and “non-professional support”. Table 2 summarizes the results in form of a diabetes app (non-) user typology, which displays the differences in social support in differing app user and non-user groups.

**Table 2:** Diabetes App (Non-)Use and Social Support – Study 1

	Non-User of Diabetes Apps		Diabetes App User		
App user type	The diabetes app non-user without interest in diabetes apps	The interested non-user of diabetes apps	The dissatisfied adopter of diabetes apps	The experienced diabetes app switcher	The consistent long-term diabetes app user
Self-management	No risk group, experienced with good perceived diabetes knowledge, mainly good self-management	Diabetes risk group, lacking diabetes knowledge or misperceptions, avoidance strategies, insufficient self-management, dangerous health behaviors	No risk group, good diabetes knowledge (educated at young age), good self-management	No risk group, very good diabetes knowledge, very good self-management, experienced, strict regimen	No risk group, specialized diabetes knowledge, intense diabetes education, good self-management, strict carb counting, active
Medical specialty of healthcare professionals (HCPs)	Mainly general practitioners	Mainly general practitioners	Diabetes specialists	General practitioners or diabetes specialists	Diabetes specialists, partly other HCPs as part of a diabetes program
Perceived physician quality	Partly incontent, or content after choosing selected physicians	Incontent, physicians not supportive (with exceptions)	Content but also seeing downsides	Content or incontent, depending on medical specialty of physician	Content, physicians as “friends”
Decision-making	Independent patient decision-making	Dependent or independent patient decision-making with dangerous health behaviors	Independent patient decision-making but listening to HCP advice, shared decision-making	Independent patient decision-making	Shared decision-making with close relationships between HCP and patient
Physician communi-cation	Partly short consultations, no engagement, patients need to ask questions to receive information	Short consultations, physicians not helpful, answer questions only (no further engagement)	Consultations also through Email/online/ calls, close relationships with intense communication, partly busy doctors	Short consultations (general practitioners), longer consultations but more expensive (specialists), partly contact through Email	Discussions similar to friends’ relationships, honesty in consultations, partly long consultations
Support group participation	Support group leader or follower	No support group participation or support group follower only	Support group follower or leader, volunteering for other patients, part of diabetes program	Support group leader or follower	Support group leader, part of a diabetes or app pilot program
Family/friend support	Managing without support or negative influences by family/friends (but perceived relevance of support)	Managing without support, rarely support by family	Involvement/ support by family only right after diagnose (beginning of the disease)	Support especially by friends, family support	Partly family support, sometimes negative family/friend influences
Interviewees (IP no., age group, diabetes type)	IP2, 56–60, T2DM IP7, 66–70, T2DM IP8, 61–65, T1DM IP9, 66–70, pre-diab (IP17, 61–65, T2DM)	IP4, 46–50, T2DM IP10, 56–60, T2DM (IP12, 61–65, T2DM) IP15, 46–50, T2DM IP20, unknown, T2DM	IP1, 16–20, T1DM IP11, 21–25, T1DM IP16, 21–25, T1DM IP18, 16–20, T1DM	IP3, 66–70, T2DM IP13, 56–60, T1DM IP21, 56–60, T1DM	IP5, 26–30, T2DM IP6, 56–60, T2DM IP14, 41–45, T1DM IP19, 31–35, T1DM

*Note*. Table based on Brew-Sam [4]

#### Diabetes app (non-) use in the sample – description of the dependent variable

App (non-) use categories ranged from “no previous use”, “(no) interest in apps”, and “(no) knowledge about existing diabetes management apps”, to “infrequent and short-term app use” to “long-term app use”. Some interview participants had never used diabetes apps for self-management before and expressed no interest in them (e.g., IP8, IP9), while others showed interest but lacked knowledge about appropriate app use (e.g., IP4, IP15). Most participants were familiar with available diabetes apps (apart from a few without any knowledge about app availability, e.g., IP12 and IP15). Reported diabetes-specific app use was almost exclusively limited to logbooks for blood glucose monitoring (e.g., DAFNE online App, MySugr, Glooko, DiabetesM) and to food databases displaying nutritional information (e.g., food database app developed by the Singaporean Health Promotion Board). Moreover, app users split into short-term users who were mostly unsatisfied with the current state of diabetes apps and had abandoned their use after a while (“adopters”, e.g., IP16, IP11), users who constantly switched apps, using several in parallel (“switchers”, e.g., IP3, IP21), and users who used one main app over a longer period of time (“long-term users”, e.g., IP5, IP6) (Table 2).

#### Professional support – physician-patient relationship

The themes derived from the interviews regarding healthcare professional support in (technology-supported) diabetes management included “medical specialty of physician related to perceived care quality”, “taking time for communication”, “actual decision-making”, “decision-making preferences”, and “inclusion of apps in physician-patient relationship”.

Physicians were mentioned as the main supervisors in diabetes care, with other healthcare professionals (e.g., dieticians, nurse educators, podiatrists, pharmacists) only partly included in care with considerable variation amongst respondents (IP2, IP3, IP6, IP7). Thus, we further focused on the physician-patient relationship.

Patients supervised by general practitioners (GPs) reported short consultations with brief physician-patient communication (“the doctor is 5-10 minutes only”, IP2, age 56–60, T2DM; “if you ask questions, they will answer... but they won’t engage you for too long”, IP8, age 61–65, T1DM), perceived GPs lacking diabetes knowledge, and a perceived lack in support (“they [physicians] are not helpful”, IP4, age 46–50, T2DM). They mostly expressed dissatisfaction with the quality of supervision by their GPs. The group of dependent patients, defined as those mainly following doctors’ instructions without taking diabetes-related decisions or being less active in decision-making (e.g., IP12, age 61–65, T2DM), mostly consulted GPs. Additionally, non-users of apps mostly consulted GPs (Table 2).

In contrast to GPs, diabetes specialists were reported as providing adequate time for support: “you got two types [of physicians]... one we call it family physician… one uh he charge you more, double [specialist]… this doctor will spend more time” (IP3, age 66–70, T2DM). Moreover, specialists sometimes developed close relationships with their patients (“he’s... more like a family friend ... than a doctor”, IP6, age 56–60, T2DM) and mostly followed shared decision-making approaches. Yet, some patients considered their physicians merely as advisors, preferring to take diabetes care decisions independently (e.g., IP1, IP3) (Table 2).

Overall, few patients reported that physicians or other healthcare professionals (nurses) talked about diabetes apps in the consultations, or shared app information with them (e.g., IP2, IP3, IP6). Some patients participated in diabetes programs (e.g., DAFNE, dose adjustment for normal eating) that included specialist supervision and an app for self-management (e.g., IP1, IP19). Apart from these specialist programs, physicians hardly used apps to communicate with their patients (sometimes Email, e.g., IP5).

#### Non-professional support – family & friends’ support

Self-management support by family and by friends fell into the categories of “managing alone”, “negative support”, “involvement only after diagnosis”, and “strong involvement”. Both app users and non-users reported receiving support by their family and friends in their diabetes management (Table 2), either after diagnosis at the beginning of self-care (e.g., IP11, IP18) or throughout the whole process of self-care (e.g., IP21, IP13). Negative support was reported when their social contacts tempted the patients towards unhealthy lifestyles, e.g., “they always say ‘never mind! Eat, just eat! Only once! You don’t eat this every day’“ (IP9, age 66–70, pre-diabetes), or when involvement of the family resulted in nagging behaviors (“whenever they are with me... when the doctor tells me something… and then after … when we go home they immediately start nagging me”, IP16, age 21–25, T1DM). Positive attitudes towards diabetes care by family and friends were mentioned as important for self-management (“the key is that they’re... not uhh... really that negative on this... they’re also very positive”, IP13, age 56–60, T1DM). Some non-users of diabetes apps said they managed their condition alone, without the involvement of family or friends (“It’s myself, nobody else... No friends, no, nobody else, it is me”, IP8, age 61–65, T1DM).

Overall, the interviews showed that aspects of professional support (style of decision-making, duration and quality of communication) related to perceptions of satisfaction and success in self-management. Moreover, this appeared to be influenced by the medical specialty of the physician supervising the patient (compare [4]). Regarding non-professional support, the support by family and friends diversely related to (technology-supported) self-care, with both positive and negative influences on self-management reported. Based on the exploratory interview results we specified the theoretically derived hypotheses *H1* and *H2* as following:

*(H1a) Supportive behaviors by the supervising physician (shared decision-making styles and supportive communication) positively predict diabetes app use for self-management.*

*(H1b) The medical specialty of the supervising physician (specialist* versus *GP) is a predictor of diabetes app use for self-management, with specialist care promoting app use to a greater extent than care by GPs.*

*(H2a) (Positive) support by the patient’s personal social network (family and friends) positively predicts diabetes app use for self-management.*

### Online survey results on social support and diabetes app use (Study 2)

In Study 2 we tested hypotheses *H1a*, *H1b*, and *H2a* using binary logistic regression. Checking for autocorrelation of all independent variables, physician decision-making and physician-patient communication were highly correlated with *r* = .772, *p <* .01. Therefore, decision-making and communication were recoded into a single variable “physician-patient relationship” [4].

Based on theoretical considerations and the interview results, we began with a binary logistic regression model that included a maximum of independent factors: physician-patient relationship, medical specialty of the physician, family/friend support, and other relevant factors from technology adoption theory [(51] and from self-management theory [7] shown to be relevant predictors of technology use for disease self-management. We then compared different models by reducing independent factors to find the model with the best fit.

A model with good fit included the factors derived from the interviews *physician-patient relationship, family/friend support, medical specialty of the physician,* as well as the additional UTAUT factors’ *perceived app potential* (performance and effort expectancy), *previous health information seeking online* (technological experience), and *age*; and the self-management factors *type of diabetes*, *length of diabetes*, *perceived health status*, *payment problems* and *insurance coverage* (socioeconomic), *blood glucose testing adherence* (self-management behaviors), *interest in innovation* (attitudes), *perceived diabetes knowledge*, *program or support group participation, and psychological empowerment*. The test of this model against the constant-only model was statistically significant, indicating that the predictors as a set reliably distinguished between diabetes app use and non-use (χ^2^(16) = 26.752, *p <* .05). Nagelkerke’s *R*^2^ = .656 indicated a high relationship between prediction and grouping (goodness-of-fit). Prediction success overall was 90% (80% for app non-use and 96% for app use). The Wald criterion demonstrated that only the family/friend support (*Wald* (1) = 5.315, *p* = .021) and the medical specialty of the consulted physician (dummy GP or specialist, *Wald* (1) = 4.014, *p* = .045) made a significant contribution to the prediction of diabetes app use. The *Exp*(*β*) value indicated that when the family/friend support was increased, the relative probability (odds ratio) that diabetes apps were used decreased with *Exp*(*β*) = .044, *β* = − 3.131. The *Exp*(*β*) value indicated that when the patients were supervised by specialist doctors the relative probability (odds ratio) that diabetes apps were used increased with *Exp*(*β*) = 9460.805, *β* = 9.155. In contrast to non-professional support (family/friends), the physician-patient relationship was not found to be a significant predictor in the model.

A model resulting after the removal of the factors *interest in innovation*, *insurance coverage*, and *program or support group participation* (due to lacking significance) showed a slightly lower prediction success with 75% (70% for app non-use and 79% for app use), but overall model significance with χ^2^(13) = 26.936, *p <* .05, and a moderate to high relationship between prediction and grouping (goodness of fit) with Nagelkerke’s *R*^2^ = .509. In this model, the Wald criterion demonstrated that the family/friend support (*Wald* (1) = 6.617, *p* = .010) and perceived health status (*Wald* (1) = 7.839, *p* = .005) made a significant contribution to the prediction of diabetes app use. Again, the *Exp*(*β*) value showed that when support by family/friends was increased, the relative probability (odds ratio) that diabetes apps were used decreased with *Exp*(*β*) = .283, *β* = − 1.261. The *Exp*(*β*) value also indicated that when the perceived health status was improved the relative probability (odds ratio) that diabetes apps were used increased with *Exp*(*β*) = 8.030, *β* = 2.083.

Further reducing the independent factors, the models showed similar results to the last model, resulting in *family/friend support* and the *perceived health status* being significant predictors of diabetes app use. A further reduction of factors decreased the model fit, yet the *medical specialty of the physician* nearly reached significance again.

Overall, after testing various models, only the family/friend support, the medical specialty of the supervising physician, and the perceived health status significantly predicted diabetes app use. Less family/friend support was likely leading to a higher chance of diabetes app use, while the use of diabetes specialists or a better perceived health status increased the chance of app use for self-management.

Despite the physician-patient relationship lacking significance for predicting diabetes app use in the models (apart from the medical specialty of the physician as a significant predictor), additional *t*-test calculations showed that the medical specialty of the physician related to the style of decision-making and the physician-patient communication. There were differences between specialists and GPs, with specialist care positively related to higher shared decision-making and better physician-patient communication as compared to GPs (Decision-making PDMstyle: specialists: *M* = 3.72, *SD* = 1.16, *n* = 37, general practitioners: *M* = 2.65, *SD* = 1.30, *n* = 22; *t* [57] = − 3.275, *p* < .01, *N* = 59; Communication PCOM: specialists: *M* = 3.82, *SD* = 1.01, *n* = 37, general practitioners: *M* = 3.13, *SD* = 1.09, *n* = 22; *t* [57] = − 2.472, *p* < .05, *N* = 59) (compare [4]).

## Discussion

In general, this two-study project found that social support from both professional and patient’s personal social networks had an influence on mobile app usage in the context of diabetes self-care.

Regarding professional support, the interviews (Study 1) showed that shared decision-making and supportive communication, including support regarding diabetes technology use, depended on the medical specialty of the physician, with patients perceiving support by diabetes specialists as more helpful than by GPs. Binary logistic regression (Study 2) confirmed these results, showing that the specialty of the physician significantly predicted the use of diabetes apps by patients, with supervision by diabetes specialists increasing the likelihood of the apps being used (as opposed to GPs). Specialist care was positively related to higher shared decision-making and better physician-patient communication. These study results conform with existing research, which points towards differences in diabetes care depending on the medical specialty of the supervising physician.

A study by Koizumi et al. [74], for example, reported that attitudes towards glucose control and self-care varied in physicians depending on their medical specialty. According to these researchers, patients’ self-management behaviors can be influenced by physicians’ beliefs and behaviors. De Berardis et al. [75] compared diabetes consultations in 125 diabetes outpatient clinics and 103 general practices for process and intermediate outcomes (frequency of examinations, HbA1c, blood pressure, and cholesterol levels) over a period of 2 years. They found significant better results for the majority of the process measures, and for cholesterol levels, in the specialized diabetes outpatient clinics as compared to the general practices. In particular, care by the same specialist in a diabetes outpatient clinic ensured better quality of care in comparison with other care options. Yet, these studies did not relate to diabetes technology use. Thus, our results add preliminary evidence that differences do not just affect traditional but also technology-supported diabetes care (e.g., diabetes app use), with specialist care promoting technology-supported self-management to a greater extent than care by GPs. Reasons for this might be found in a connection between more extensive knowledge about diabetes and mHealth apps, and longer consultation duration afforded by specialists. It is also possible that government initiatives to promote mHealth have been promoted more extensively with specialists. However, given that we did not interview healthcare professionals, these are assumptions that need to be examined in further research.

Examining the use of technology in physician-patient relationships further, other studies generally discussed the usefulness of health apps for physician-patient interaction, for example, in shared decision-making [36]. Abbasgholizadeh et al. [36] reported mixed results for technology-supported physician-patient interactions; for example, better accessibility to data, improved efficiency for the physician, real-time connectivity, or remote decision-making on the one hand, but diminished quality of care through overuse of mHealth, increased health disparities due to lacking mHealth access, or lack of mHealth regulation, on the other hand.

In terms of non-professional support, the patients’ personal network was perceived as either facilitating or hindering the use of mobile technology for self-management, depending respectively on the presence of supportive or unsupportive behaviors (Study 1). Moreover, support by this non-professional network significantly predicted patients’ use of diabetes apps, with lower family and friend support increasing the likelihood of using diabetes apps (Study 2). Potential explanations for the negative relationship could be that certain patient groups are substituting technology to compensate for a lack of perceived or actual social support (e.g. [76]). There is an emergent research field examining loneliness and Internet use [77, 78], with studies suggesting that technology can offer a solution for chronically ill patients who feel isolated with their disease and who search for a (technological) solution to overcome their lack of social connection [79]. As mentioned earlier, support by relatives and friends is not necessarily always positive, for example, when unsupportive behaviors or miscarried helping occur [16, 22, 26]. Thus, support can be sought elsewhere, in this case, via technology substitution. Conversely, high social support by family and friends might nullify the need for additional technology to support self-care, which might explain why higher social support accompanies less technology use. In general, our results support the extant literature showing that the patients’ social network influences attitudes towards innovation, which influence innovation adoption behaviors in return [80].

### Future research

Future research needs to examine aspects of social support influencing mHealth use, also comparing the influence of professional versus non-professional support on mHealth use. Apart from the social influence of health professionals and of family and friends on mHealth use as studied here, previous studies show that “peer” patients also play an important role as sources of information and of empowerment for patients [38, 81]. Thus, firstly, follow-up research should include the role of patient peers. Secondly, the investigation can be extended from physicians to other healthcare professionals involved (e.g., nurse educators). Thirdly, mHealth studies should extend their theoretical foundations beyond technology adoption theories. There are few theories applied in mHealth research, which mainly relies on traditional behavior change theories [82, 83]. To investigate factors influencing and predicting technology use for self-management, a broader theoretical foundation needs to be available, overcoming the limitations of a very narrow range of theories. Fourthly, even though the socioeconomic and demographic factors included in the regression analysis (e.g., age, insurance coverage) did not predict the use of diabetes apps, it has to be further examined if these factors have an impact on the results, like the association between the specialty of the physician and the use of diabetes technology, or the negative association between non-professional support and technology use. Similarly, and fifth, an influence of app characteristics and app quality on diabetes app use is to be expected and follow-up research has to examine how these app characteristics interrelate with other factors influencing app use for diabetes self-management, including aspects of social support.

### Implications for research, policy and practice

While our study focused on diabetes apps, these results can equally inform mHealth research in other health domains involving self-management, including various chronic conditions such as heart diseases, obesity, asthma, stroke, cancer, arthritis, hypertension, multiple sclerosis, etc. [84, 85]. Even though there are specific characteristics for each disease, general knowledge about technology use for self-management can be helpful across health conditions to design technology accordingly.

For diabetes policy and practice, the study results suggest that there are notable differences in patient groups regarding diabetes app use, with diabetes patients being a heterogeneous group with varying needs [86]. Segmentation theory needs to be used to tailor diabetes app characteristics specifically for subgroups with varying preferences and needs regarding technology (also considering socioeconomic, cultural, and demographic differences). Moreover, influencing factors need to be considered when designing technology for diabetes care, like the influence of aspects of social support and social influence [87]. Both healthcare professional supervision and family/friend support can influence technology-supported self-management. In particular, the care provided by GPs was perceived as unsatisfactory by most diabetes patients in the Study 1 sample. This resulted in less mobile technology use as compared to specialist supervision, which deficiency should be addressed to achieve equal care for all patients. The inclusion of relatives in (technology-supported) self-care should be further investigated depending on patient preferences and the usefulness of support provided (to avoid misguided support).

### Study limitations

Study limitations included the small sample size of the online survey, which limited sophisticated multi-variate data analysis [4] and broad generalizability. Considerable effort was undertaken to achieve a larger sample size (extension of the field phase, seeking contact with official care organizations, seeking cooperation with healthcare professionals, repeated invitations for survey participation), yet without success. Singaporean citizens suffering from diabetes receive a large amount of invitations for study participation since the government declared “the war against diabetes” in 2016 [88], so these are a prized yet scarce resource for research. Due to the cross-sectional nature of the study design, final conclusions about causality could not be drawn. Additionally, due to the recruitment method a certain degree of self-selection was unavoidable (over-representation of support group members and educated participants). The sample was therefore not representative for all Singaporean individuals with diabetes; however, we managed to include participants of the main Singaporean cultural backgrounds in the sample (Chinese, Malay, Indian). These three groups comprise 97% of Singaporeans [89]. Finally, triangulation of the study findings with results on diabetes app features and quality [4, 5] was not undertaken here due to length limitations.

## Conclusion

Our study results indicated that aspects of both professional and non-professional support have an impact on the use of mobile self-management technology by diabetes patients. However, the results also showed that the effects of support are not always positive for technology use. This leads to the conclusion that social support in diabetes self-management needs to be considered and analyzed in a differentiated manner when looking into social support as a predictor of technology use.

## Data Availability

The (anonymized, non-identifiable) datasets used and/or analyzed during the current study are available from the corresponding author on reasonable request. Additional information on the methodology (questionnaires, survey) can be found online [63].

## Abbreviations

T1DM/T2DM: Type 1/Type 2 Diabetes Mellitus
GP: General practitioner
HCP: Healthcare professional
IP: Interview participant
UTAUT: Unified Theory of Acceptance and Use of Technology
DAFNE: Dose Adjustment for Normal Eating
